# Semaphorin-3D and Semaphorin-3E Inhibit the Development of Tumors from Glioblastoma Cells Implanted in the Cortex of the Brain

**DOI:** 10.1371/journal.pone.0042912

**Published:** 2012-08-24

**Authors:** Adi D. Sabag, Julia Bode, Dorit Fink, Boaz Kigel, Wilfried Kugler, Gera Neufeld

**Affiliations:** 1 Cancer Research and Vascular Biology Center, The Bruce Rappaport Faculty of Medicine, Technion, Israel Institute of Technology, Haifa, Israel; 2 Department of Pediatric Hematology and Oncology, University of Goettingen, Goettingen, Germany; The Chinese University of Hong Kong, Hong Kong

## Abstract

Class-3 semaphorins are secreted axon guidance factors. Some of these semaphorins have recently been characterized as suppressors of tumor progression. To determine if class-3 semaphorins can be used to inhibit the development of glioblastoma-multiforme tumors, we expressed recombinant sema-3A, 3B, 3D, 3E, 3F or 3G in U87MG glioblastoma cells. Sema3A and sema3B expressing cells contracted and changed shape persistently while cells expressing other semaphorins did not. Sema3A and sema3F differed from other semaphorins including sema3B as they also inhibited the proliferation of the cells and the formation of soft agar colonies. With the exception of sema3G and sema3B, expression of these semaphorins in U87MG cells inhibited significantly tumor development from subcutaneously implanted cells. Strong inhibition of tumor development was also observed following implantation of U87MG cells expressing each of the class-3 semaphorins in the cortex of mouse brains. Sema3D and sema3E displayed the strongest inhibitory effects and their expression in U373MG or in U87MG glioblastoma cells implanted in the brains of mice prolonged the survival of the mice by more then two folds. Furthermore, most of the mice that died prior to the end of the experiment did not develop detectable tumors and many of the mice survived to the end of the experiment. Most of the semaphorins that we have used here with the exception of sema3D were characterized previously as inhibitors of angiogenesis. Our results indicate that sema3D also functions as an inhibitor of angiogenesis and suggest that the anti-tumorigenic effects are due primarily to inhibition of tumor angiogenesis. These results indicate that class-3 semaphorins such as sema3D and sema3E could perhaps be used to treat glioblastoma patients.

## Introduction

Glioblastomas are the most common primary tumors arising in the central nervous system. However, despite enormous efforts the median survival time after initial diagnosis of their most aggressive form, glioblastoma multiforme (GBM), is still only 50 weeks [1]. GBM, the most malignant form of infiltrating astrocytoma, can evolve from a lower grade precursor tumor or can evolve as a highly malignant tumor from the outset. GBMs form highly vascularized tumors expressing elevated levels of numerous angiogenic factors such as vascular endothelial growth factor (VEGF), fibroblast growth factor-2 and interleukin-8 [2]. Grade II infiltrating astrocytomas have a vessel density similar to that of normal brain but as astrocytomas progress to grade III the vascular density increases. GBM grade IV tumors often display high levels of microvascular hyperplasia, particularly in regions adjacent to necrosis in which the cells are hypoxic and over-express VEGF. It is possible that the accelerated angiogenesis noted in the transition to GBM is a key factor in rapid tumor growth and clinical progression since the survival of patients with grade III astrocytomas that lack microvascular hyperplasia is significantly longer.

The neuropilin-1 (np1) and the neuropilin-2 (np2) receptors were characterized as receptors for axon guidance factors of the class-3 semaphorin (sema3) family [3]. It was subsequently realized that neuropilins are also expressed by endothelial cells and by many types of cancer cells [4] and that they participate in the transduction of pro-angiogenic signals induced by angiogenic factors such as VEGF and hepatocyte growth factor/scatter factor (HGF/SF) [5]–[8]. Most of the class-3 semaphorins, with the exception of sema3E which binds to the plexin-D1 receptor [9], bind to one of the two neuropilins or to both [4]. Neuropilins form functional semaphorin receptors by associating with members of the plexin receptor family [10], [11]. The semaphorins sema3B and sema3F were characterized as tumor suppressor genes [12], [13] suggesting that they may function as anti-angiogenic and anti-tumorigenic factors. Indeed, several class-3 semaphorins including sema3A, sema3B, sema3E, and sema3F have by now been characterized as anti-angiogenic agents [14]–[19].

Based upon these earlier observations we wondered if class-3 semaphorins, which have been reported to be down regulated during the transition of tumors from the dormant to the angiogenic state [17], may find use as inhibitors of glioblastoma development and progression. We show that expression of several types of recombinant semaphorins in U87MG and U373MG GBM derived tumor cells inhibits tumor development following their implantation in the cortex of mouse brains and prolongs significantly the survival of these mice as compared to the survival of mice that received control cells.

## Results

### The effect of class-3 semaphorin expression on the morphology and proliferation of U87MG cancer cells

**Figure 1:**
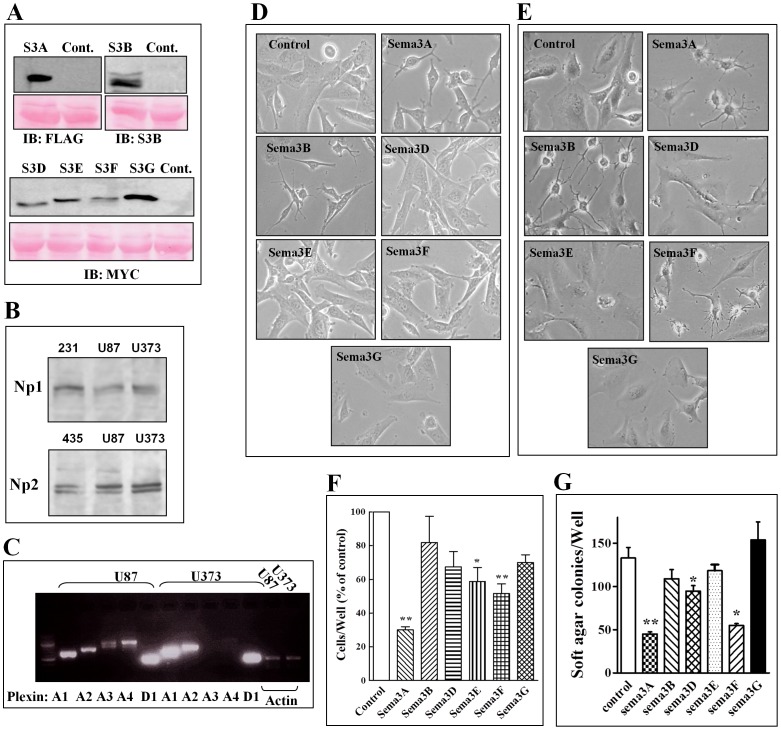
The effect of the expression of various class-3 semaphorins on the morphology and proliferation of U87MG cells: (A) Lentiviruses containing the full length cDNAs encoding six semaphorins (S3A-G) or empty control lentiviruses (Cont.) were used to infect U87MG cells. Sema3A has a FLAG epitope tag, S3B has no tag and the rest of the semaphorins were labeled with a myc epitope tag. The presence of the designated recombinant semaphorins in aliquots of serum free medium derived from equal numbers of U87MG cells (5×10^5^ cells/well) expressing the different sema3s. The serum free medium was conditioned for 48 h and 40 µl aliquots were then assayed for the presence of the designated semaphorins by western blot analysis (upper panels). To verify equal protein loading the blot was also stained with ponceau-red. Shown is a ponceau-red stained 60 KDa protein found in the serum free medium (B) Cell lysates were prepared from U87MG or U373MG cells as described. The content of neuropilin-1 (Np1) and neuropilin-2 (Np2) in aliquots of cell lysates (50 µg protein) were determined by western blot analysis as described. Cell lysates from neuropilin-1 expressing MDA-MB-231 cells and neuropilin-2 expressing MDA-MB-435 cells served as positive controls [14]. (C) Reverse PCR was performed using total RNA isolated from U87MG (U87) or U373MG (U373) cells in order to detect the expression of mRNA encoding PlexinsA1–A4 (A1–A4) and PlexinD1 (D1) as described. Expression of β-actin (Actin) mRNA was used as a loading control. (D) U87MG cells infected with control lentiviruses (Control) or with lentiviruses directing expression of the designated semaphorins were cultured in cell culture dishes and photographed. (E) U87MG cells were seeded (1×10^4^ cells/well) in 12 well plates. The next day cells were incubated with conditioned mediums derived from HEK293 cells expressing the various recombinant semaphorins. Cells were photographed after a 30 minute long incubation. (F) U87MG cells infected with empty lentiviruses (Control) or with lentiviruses directing expression of the designated recombinant semaphorins were seeded in triplicate in 24 well dishes and their proliferation measured as described in materials and methods. Each bar represents the average of three independent experiments each of which was done in triplicates. (G) Single cell suspensions of U87MG cells infected with empty control lentiviruses (Control) or U87MG cells expressing the designated class-3 semaphorins were seeded in triplicates in soft agar as described in materials and methods. Colonies were allowed to develop for 21 days. Colonies were then stained with crystal violet and microscopic fields photographed. Colonies with a diameter exceeding 100 µm were counted. The average number of colonies in triplicate wells was calculated. Shown is a representative experiment from two experiments which produced similar results.

U87MG cells are derived from a human GBM tumor and are commonly used to study brain cancer since they form tumors quickly and with high frequency when implanted subcutaneously in immune deficient mice. To find out whether class-3 semaphorins can be used to inhibit the development of brain tumors we expressed several class-3 semaphorins (sema-3A, 3B, 3D, 3E, 3F and 3G) in U87MG cells using previously described lentiviral expression vectors [14]. In the cases of sema3B and sema3E we did not use the cDNA encoding the native semaphorins but previously described cDNAs encoding furin resistant point mutants of these semaphorins [19], [20]. The various recombinant human semaphorins were epitope tagged at their C-termini with a myc epitope tag except for sema3A which contained a FLAG epitope tag and sema3B which was untagged. The expression levels of these recombinant class-3 semaphorins were examined in cell lysates (data not shown) and in conditioned medium derived from pools of infected U87MG cells (Fig. 1A). The semaphorins sema3A, sema3B and sema3G were found in the conditioned mediums in relatively high concentrations while the amounts of sema3D, sema3E and sema3F found in the conditioned medium of the infected U87MG cells seemed somewhat lower (Fig. 1A). When the effects of the expression of the different recombinant semaphorins on the morphology and behavior of the infected U87MG cells were examined, we found that expression of sema3A and sema3B had a profound persistent effect on the morphology of the U87MG cells, and caused the cells to contract (Fig. 1D). In contrast, Sema3F expressing cells transiently contracted following sema3F expression but the morphological effect was subsequently lost while expression of other semaphorins did not affect the morphology of the cells (Fig. 1D). In agreement with these results, addition of exogenous sema3A, sema3B or sema3F to un transfected U87MG cells but not addition of other semaphorins also induce contraction of the cells (Fig. 1E). The lack of response to sema3E, sema3D and sema3G was observed even though both neuropilins as well as all of the Type-A plexins and plexin-D1 were found to be expressed in U87MG cells (Fig. 1B & 1C). The morphological effects were accompanied in the cases of sema3A, but not in the case of sema3B, by significant inhibition of cell proliferation and anchorage free growth (Fig. 1F & 1G). Interestingly, even though expression of sema3F did not result in persistent morphological changes, its expression in U87MG cells nevertheless significantly inhibited their proliferation as well as their ability to form colonies in soft agar suggesting that the morphological effects of the semaphorins are not necessarily correlated with their effects on cell proliferation (Fig. 1F & 1G).

### The effect of class-3 semaphorins on the development of subcutaneous tumors derived from GBM cancer cells

**Figure 2:**
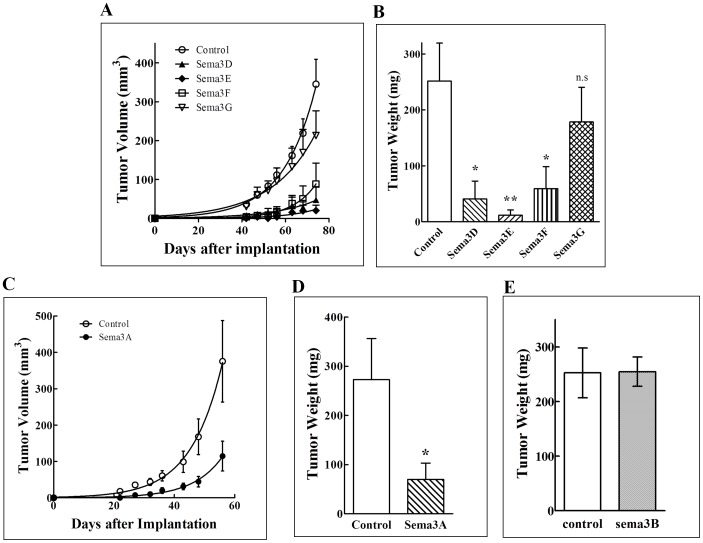
The effect of class-3 semaphorins on the development of subcutaneous tumors derived from u87 mg glioblastoma cells: U87MG cells infected with control lentivirus (control) or infected with lentiviruses directing expression of the designated semaphorins (Sema3X) were implanted subcutaneously in balb\c nu/nu mice as described. (A, C) The average volume of the developing tumors was measured as described. (B, D, E) The average weight of the tumors was determined at the end of the experiment as described.

To determine if the expression of class-3 semaphorins in U87MG cells is accompanied by effects on tumor formation, we implanted U87MG cells expressing different recombinant class-3 semaphorins subcutaneously in nu/nu balb/c nude mice. We tested six of the seven class-3 semaphorins in these experiments. Interestingly, even though Sema3D and sema3E inhibited either proliferation or anchorage free growth of the U87MG cells in-vitro much less effectively than sema3A (Fig. 1F & 1G), they still inhibited subcutaneous tumor development as effectively as sema3A if not more effectively (Figs. 2B & 2D). These results suggest that the main inhibitory effect of these two semaphorins is directed at the stromal cells of the tumor microenvironment rather than at the tumor cells themselves. The np2agonist sema3F also inhibited tumor formation effectively although somewhat less effectively than sema3E and sema3D (Fig. 2B). Surprisingly, we did not observe inhibition of tumor development following the expression of sema3G, which like sema3F binds to np2, or following expression of sema3B which binds to both of the neuropilins (Fig. 2B & 2E).

**Figure 3:**
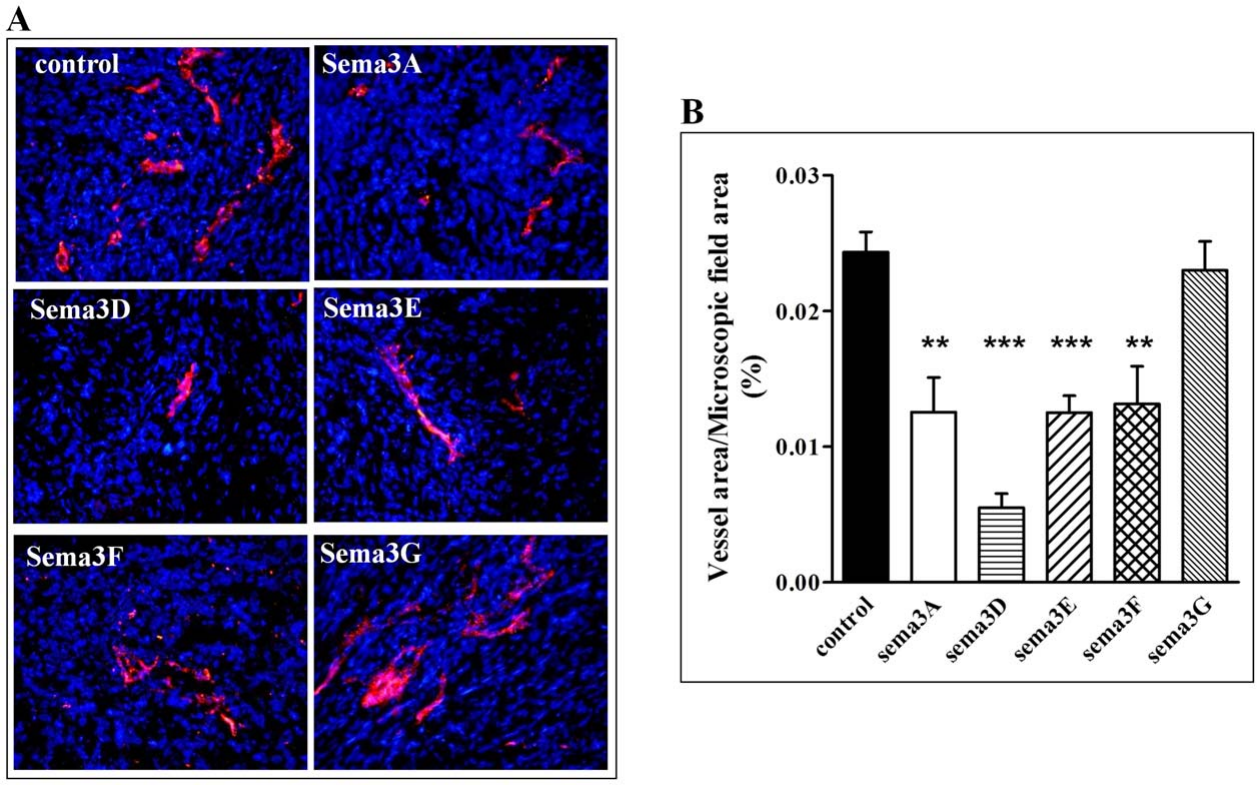
The effect of the expression of class-3 semaphorins on the microvascular density of subcutaneous tumors derived from U87MG glioblastoma cells: Tumors excised from mice injected subcutaneously with U87MG cells infected with empty control lentiviruses (control), or lentiviruses directing expression of the designated class-3 semaphorin were embedded in OCT and frozen as described in materials and methods. Frozen sections (20 µm) were stained with anti CD-31 antibody (red) and nuclei were stained with DAPI (blue), as described in materials and methods. (**A**) Representative pictures (20×) of microscopic fields taken from tumor areas in which the density of blood vessels was highest. Pictures were taken using a fluorescent microscope. (**B**) The area of CD-31 stained blood vessels in fields of equal area was quantified as described in materials and methods.

These observations suggest that inhibition of angiogenesis is likely to represent the major mechanism by which class-3 semaphorins inhibit the development of tumors from U87MG cells implanted subcutaneously. In agreement with this hypothesis we have found that tumors that developed from cells expressing the most potent inhibitors of tumor development, sema3A, sema3D, sema3E and sema3F, contained significantly reduced concentrations of blood vessels while sema3G which did not inhibit tumor development significantly also failed to reduce the density of blood vessels in tumors (Fig. 3).

### The effect of class-3 semaphorins on the development of tumors from GBM cells implanted in the brain cortex

**Figure 4:**
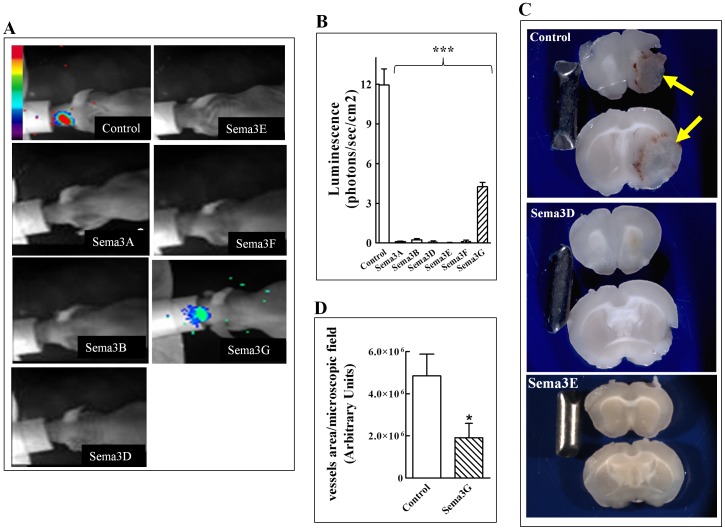
The effect of class-3 semaphorins on the development of tumors from U87MG glioblastoma cells implanted in the brain cortex: (**A**) U87MG cells infected with control lentivirus or infected with lentiviruses directing expression of six different semaphorins (Sema3x) were implanted in the brain cortex of CD1 nu/nu mice (8 mice/group) as described. Tumor growth was followed in *vivo* using bioluminescence imaging following administration of D-luciferin as described. Shown are representative pictures of pseudo colored overlay images of the bioluminescence signal intensity superimposed on mice. The pictures were taken 22 days after the implantation of the tumor cells. (**B**) Quantification of luminescence in mice in which control or semaphorin expressing tumor cells were implanted as described above. Luminescence was measured 22 days after the implantation of the cells as described in materials and methods. Each bar represents the average luminescence from 8 different mice. (**C**) Brains from mice injected with U87MG cells infected with empty control lentiviruses (Control), or lentiviruses directing expression of sema3D or sema3E were sliced transversely and the slices were photographed. A large brain tumor is seen in the control (yellow arrows) whereas no tumors can be detected in brains in which sema3D or sema3E expressing U87MG cells were implanted. (**D**) Tumor sections from the brain cortex of mice implanted with U87MG cells infected with empty control lentiviruses (Control), or lentiviruses directing expression of sema3G (sema3G) were sectioned perpendicularly at the injection site, fixed in formalin, and embedded in paraffin. Paraffin sections (4 µm) were stained with anti-vWF using a fluorescent microscope as described in materials and methods. Microscopic fields were photographed and the vessel area per microscopic field was calculated as described in materials and methods.

Since spontaneous glioblastoma tumors develop in the brain we determined if the expression of the semaphorins would have similar inhibitory effects on tumor formation from U87MG cells implanted in the cortex of the brain. To monitor tumor development by non-invasive methods, we used U87MG cells expressing recombinant firefly luciferase. Cells infected with lenti-viruses directing expression of different semaphorins in addition to luciferase were injected into the brain cortex of CD-1 immune deficient mice (7.5×10^4^ cells per mouse). To follow tumor development luciferin was injected into the peritoneum of mice and a VisiLuxx Imager (Visitron Systems) was used to detect light emission from luciferase expressing cells. After three weeks light emitting focuses could be detected in the brains of all of the surviving mice implanted with control cells or with sema3G expressing cells, while none of the mice that received any of the other semaphorin expressing cell types developed discernable tumors (Figs. 4A & 4B). When whole brain sections were examined at the end of the experiment tumors could easily be detected by gross examination in the brains of all control animals (Fig. 4C). Tumors were also detected in brains of animals implanted with cells expressing sema3G (not shown) but not in brains of animals implanted with cells expressing sema3A, sema3B, sema3D, sema3E or sema3F. Representative brain slices from animals implanted with control cells and of animals injected with sema3D and sema3E are shown in Fig. 4C. Although tumors did develop in the brains of animals implanted with cells expressing sema3G, the development of these tumors in the brain cortex, as opposed to their development following subcutaneous injection, was significantly retarded (Fig. 4A & 4B). When we compared the microvascular density in tumors that developed from control cells with that of tumors that developed from sema3G expressing cells we found that the microvascular density was significantly lower in tumors that developed in the brain cortex from sema3G expressing cells (Fig. 4D). These results suggest that class-3 semaphorins inhibit angiogenesis more effectively in the brain than in the subcutaneous microenvironment.

In these experiments we identified 5 different class-3 semaphorins as potentially potent inhibitors of glioblastoma development. These were sema3F, sema3A, sema3B, sema3E and sema3D. As a result of the combined subcutaneous and intra-cerebral tumor experiments we concluded that sema3E and sema3D may represent the most potent inhibitors, so we used only these two semaphorins in subsequent experiments. Sema3D binds to both np1and np2while sema3E employs plexin-D1 as its receptor [4] suggesting that these two semaphorins use somewhat different pathways to exert their anti-tumorigenic effects.

**Figure 5:**
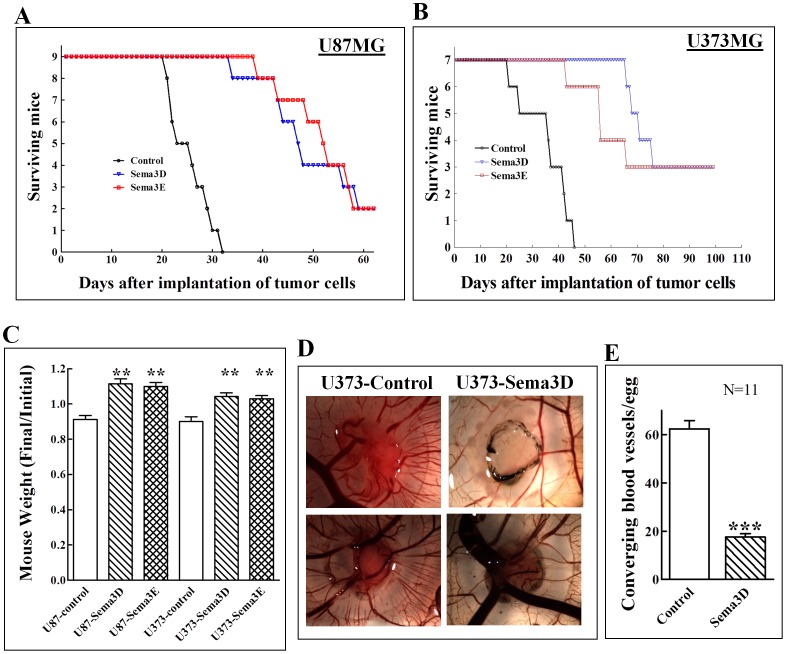
The effect of sema3E and sema3D expression in glioblastoma cells on the survival of tumor bearing mice: (**A**) U87MG cells infected with empty lentiviruses (Control) or with lentiviruses directing expression of sema3D or sema3E were implanted in the brain cortex of mice. Nine mice were included in each group and the effect of the expression of the semaphorins on their survival following implantation measured. (**B**) U373MG cells infected with empty lentiviruses (Control) or with lentiviruses directing expression of sema3D or sema3E were implanted in the brain cortex of mice. Seven mice were included in each group and the effect of the expression of the semaphorins on their survival following implantation measured. (**C**) The weight of the mice that participated in the experiments shown in panels C and D was measured on the day at which the cells were implanted and at the end of the experiment or following the death of the mice. Shown is the average ratio between body weight at the start of the experiment and at the end, for each of the groups of mice. (**D**) Control U373MG cells or U373MG cells expressing sema3D were implanted on the chorioallantoic membrane of chick embryos as described in materials and methods. The resulting tumors were photographed after 7 days. (**E**) Blood vessels converging on tumors that developed on the chorioallantoic membrane of chick embryos from control (11 eggs) or sema3D expressing U373 cells (11 eggs) were counted. Statistical analysis was performed using the unpaired data with unequal variance student's T-test.

To determine the effects of sema3E and sema3D expression in glioblastoma cells on the survival of tumor bearing mice, we implanted control U87MG cells or cells expressing either sema3D or sema3E in the brain cortex of mice and monitored tumor development and mouse survival. As in the previous experiment, we found that expression of sema3D or sema3E almost completely inhibited tumor development from cells implanted in the brain cortex of mice (data not shown). This was reflected in the effects that the expression of both semaphorins had on mouse survival. Of the mice that were implanted with control cells 50% died within 25 days after implantation. In contrast, 47 days were required until 50% of the mice that were implanted with sema3D expressing cells died and 54 days until 50% of the mice that received sema3E expressing cells died (Fig. 5A). Interestingly, it was not clear in this case if the mice died due to tumor development at all since staining of brain sections with antibodies directed against luciferase detected very few labelled cells (data not shown). Between days 40 and 50, four mice from each of the groups inoculated with sema3E or sema3D expressing cells also developed eye problems and died a few days later. However, in these mice too we could not detect tumors and we estimate that these problems were not directly associated with semaphorin expression by the implanted cells.

To make sure that these effects of the semaphorins were not limited to U87MG cells, we performed a similar experiment using U373MG glioblastoma cells. These cells did not respond by contraction to any of the semaphorins (data not shown). Here too, expression of sema3E and sema3D in the tumor cells significantly prolonged the survival of the mice. Indeed, 40% of the mice did not die at the time at which we terminated the experiment (100 days) (Fig. 5B). Of the 14 mice that were inoculated with either sema3E or sema3D expressing cells only one from each group developed a brain tumor while all the mice that received control cells had tumors. Furthermore, regardless of whether the mice received U87MG or U373MG cells, control mice that received cells infected with empty vector lost on average 10% of their body weight while mice that received semaphorin expressing cells gained between 10 to 20% of their body weight (Fig. 5C) suggesting that the expression of sema3D and sema3E rescues mice with high efficiency and is not associated with significant toxicity.

Expression of sema3D and sema3E in U87MG cells inhibited cell proliferation much less than it inhibited the growth of tumors derived from such grafted cells. Sema3A, sema3B, sema3E and sema3F function as potent anti-angiogenic factors [15], [18], [19], [21]. It is thus likely that a major part of the anti-tumorigenic effects of sema3D and sema3E is mediated through inhibition of tumor angiogenesis. However, since neither U87MG cells nor U373MG cells expressing these semaphorins formed brain tumors we could not determine if inhibition of angiogenesis represents the major mechanism by which these semaphorins inhibited the formation of brain tumors. Sema3D also displayed very potent inhibitory effects (Fig. 4A & 4B) and also inhibited relatively poorly the proliferation of the tumor cells, but its anti-angiogenic potential had not been determined yet (Fig. 1F). We therefore determined if sema3D displays anti-angiogenic properties using the chick chorioallantoic angiogenesis assay [22]. We grafted U373MG cells infected with control viruses or viruses directing expression of sema3D on the chorioallantoic membrane of chick embryos. It could be readily seen that small blood vessels converge on the small tumors that formed from the control cells. In contrast, blood vessels were not observed to converge on tumors formed from sema3D expressing cells (Fig. 5D & 5E). In addition, sema3D reduced the microvascular density in tumors that formed subcutaneously from sema3D expressing U87MG cells (Fig. 3). It therefore seems that sema3D, like other class-3 semaphorins, displays anti-angiogenic properties.

## Discussion

Class-3 semaphorins, such as sema3A, sema3B, sema3F have been previously characterized as natural tumor suppressors and there are indications that sema3E also may function as a natural tumor suppressor [12], [13], [17], [23], [24]. Previous work has also shown that these semaphorins also function as potent inhibitors of angiogenesis [15], [19], [21], and that the expression of these semaphorins as well as additional class-3 semaphorins in several types of breast cancer derived tumor cells can inhibit the growth of tumors following the subcutaneous implantation of these cells [14].

To determine if class-3 semaphorins have the potential to be used as drugs for the treatment of brain cancer we expressed all the recombinant semaphorins, with the exception of sema3C, in U87MG glioblastoma derived cells. Sema3A, sema3B and sema3F induced contraction of the cells which in the case of sema3A and sema3B persisted as a permanent morphological change. In the case of sema3A the morphological change was also accompanied by inhibition of cell proliferation, but in the case of sema3B it was not. These observations suggest that persistent cytoskeletal changes are not translated inevitably into changes in cell proliferation rates.

When these class-3 semaphorin expressing U87MG cells were implanted subcutaneously in immune deficient mice, we found that all the semaphorins tested, with the exception of sema3G and sema3B inhibited significantly the formation of tumors from the grafted cells. Sema3E and sema3D seemed to be the most effective inhibitors and abrogated almost completely tumor development in spite of their marginal effect on cell proliferation in-vitro. Further examination of the tumors revealed that semaphorins that inhibited effectively tumor development also efficiently reduced the concentration of tumor associated microvessels. The anti-tumorigenic effects exerted by the various semaphorins upon the implantation of the semaphorin expressing U87MG cells in the brain cortex were in general more potent that the effects observed following subcutaneous implantation, and even sema3G, a semaphorin that did not inhibit the development of tumors from subcutaneously implanted cells, inhibited significantly the development of tumors from cells implanted in the brain cortex. The reason for this difference may be the result of differences between the microenvironment of the brain and the subcutaneous microenvironment. However, a simpler explanation could be that the much smaller number of cells that were implanted in the brain was concentrated in a very small volume. This could result in higher local concentrations of semaphorins and thus in more effective inhibition. However, this cannot explain why sema3B turned out to be a very effective inhibitor of tumor development in the brain and a very poor inhibitor in the subcutaneous microenvironment. This puzzling observation will need to be examined in greater depth in the future.

It was previously reported by us and by others that U87MG cells endogenously express several types of class-3 semaphorins including sema3A, sema3B, sema3C and sema3F [25], [26]. It was reported that endogenous sema3A expressed by U87MG cells promotes the dispersal of the tumor cells as a result of inhibition of cell adhesion [25]. These observations suggested that sema3A is a promoter of tumor progression rather than an inhibitor. The difference between these results and ours may perhaps also be explained by the increased concentrations of sema3A that are produced by the cells as a result of the ectopic expression. These hypotheses will also need to be examined in greater depth in the future.

Since sema3D and sema3E seemed to be the most potent inhibitors of tumor formation from U87MG cells, we extended these in-vivo studies to determine if the expression of these semaphorins would also inhibit tumor development from another GBM derived cell line. Indeed, expression of recombinant sema3D and sema3E in U373MG cells also inhibited very strongly the development of tumors from these cells following their implantation in the brain cortex, suggesting that the effects of the semaphorins on the development of brain tumors may be general. Remarkably, in both the U87MG and in the U373MG experiments the expression of sema3D and sema3E had a very strong survival promoting effect on the mice. In most of the mice in which we implanted sema3E or sema3D expressing U87MG or U373MG cells we could not detect any tumors at all even after more than 60 days and most of the mice that did not survive in the groups that were grafted with the semaphorin expressing tumor cells died of unknown reasons rather than because of brain tumors. These results suggest that sema3D and sema3E have the potential to be used as effective drugs for the treatment of brain cancer.

## Materials and Methods

### Ethics Statement

All the animal work was conducted according to relevant national and international guidelines. Animal studies in which cells were implanted subcutaneously or intra-cranially were conducted both in the University of Goettingen and in the Technion, Israel Institute of technology. The Goettingen permissions to conduct these experiments were 33.14.42502-06-041/08 and 33.9.42502-04-058/09. The Technion permission from the institutional animal welfare committee was IL-095-10-2007.

### Materials

Goat anti-firefly luciferase polyclonal antibody (Abcam, Cambridge, UK) or rabbit anti-human polyclonal F8 (von Willebrand-Factor) (Invitrogen Molecular Probes; Darmstadt; Germany).

The antibodies used were: Anti c-myc (Santa Cruz, sc-40), Flag (Sigma, F1804), sema3B (Santa Cruz, sc-21204-R), Np1 (Santa Cruz, sc-7239), Np2 (Santa Cruz, sc-13117), Firefly Luciferase (Abcam, ab81823), Donkey anti goat Alexa Fluor 488 (Invitrogen, A11055), Goat anti rabbit polyclonal Alexa Fluor 594 (Invitrogen, A11012).

### Cell lines

HEK-293T cells were cultured in DMEM containing 4.5 mg/ml glucose, supplemented with 10% FCS and antibiotics. U87MG cells were cultured using MEM-EAGLE EARLE's medium with non essential amino acids, supplemented with 10% FCS, 1 mM Sodium Pyruvate and antibiotics. U373MG cells were cultured using RPMI-1640 medium, supplemented with 10% FCS and antibiotics. All cell lines were obtained from the ATCC repository. Puromycin, 2 µg/ml (Sigma) or blasticidin, 20 µg/ml (InvivoGen) were used to select infected cells.

### Generation of recombinant lentiviruses directing expression of semaphorins and luciferase and infection of cells

Class-3 semaphorin encoding cDNAs were sub-cloned into the NSPI-CMV-myc-his lentiviral expression vector, as previously described [27]. The myc epitope tag was added in frame upstream to the stop codons of the cDNAs of sema3D, sema3E, sema3F and sema3G. A FLAG epitope tag was added upstream to the stop codon of sema3A. We used a previously described lentiviral vector encoding a sema3B furin resistant mutant [19]. This sema3B mutant is referred to as sema3B throughout for convenience. Lentiviruses directing expression of these cDNAs were produced in HEK293 cells and used to infect target cells. Stably infected cells were isolated using puromicin selection as previously described [14]. The cDNA encoding firefly luciferase was subcloned into the pLenti6-V5/Dest plasmid (InvitroGen) which was then used to generate lentviruses [14]. Cells stably expressing luciferase were then selected using blasticidine.

### Western Blot analysis

Cell lysis and western blot analysis of cell lysates and of serum free conditioned medium were performed as described previously [19]. Densitometry analysis was preformed using MultiGauge software (Fujifilm).

### Reverse PCR

RNA was extracted using the PerfectPure RNA Cultured Cell Kit (5 Prime). cDNA was synthesized from 1 µg of total RNA, using the Verso cDNA Synthesis Kit (Thermo Scientific, Lafayette, CO) and random hexamers. cDNA PCR amplifications were carried out for 35 cycles in the case of plexins and 15 cycles in the case of actin using the following oligonucleotide pairs:

Plexin-A1: 5′-ctgctggtcatcgtggctgtgct 5′-gggcccttctccatctgctgcttga.

Plexin-A2: 5′-gtgcccaccaactgtgcctgtcctg 5′-tcagcgatgatgtattcccctggga.

Plexin-A3: 5′-tcttgctctcgaggttcttct 5′-acatgccaagtgatcaacgac.

Plexin-A4: 5′-gtccatctaccagggcttct 5′-ctggatgtaggactcggtga.

β-actin: 5′-tgacggggtcacccacactgtgcccatcta 5′-ctagaagcattgcggtggacgatggaggg.

### Cell Proliferation

U87MG cells were seeded in triplicate in growth medium containing 0.1% FCS in 24 well dishes (10^4^ cells/well). Adherent cells were trypsinized and counted following attachment and after 72 hours, using a coulter counter as described [14]. The ratio between these two counts in the case of the control cells was taken as 100% and the effects of the expression of the semaphorins on proliferation is presented as a percentage of this value. The expression of the semaphorins did not affect seeding efficiency which did not vary by more then 10% between different wells.

### Endothelial cells repulsion assay

Repulsion assays were carried out as previously described [28].

### Formation of subcutaneous tumors from U87MG cells

Cells expressing semaphorins, or control cells infected with empty lentiviral vectors, were implanted subcutaneously (5×10^6^/mouse) in 4–6 weeks old nu/nu balb/c mice (7 mice/group) (Harlan laboratories). Tumor development was monitored and the tumors were excised and weighted at the end of the experiment as previously described [14].

### Intracerebral implantation of U87MG and U373MG cells

U87MG or U373MG Cells expressing semaphorins, or control cells infected with empty lentiviral vector were harvested, washed, counted and adjusted to 7.5×10^4^ in 5 µl of PBS. For the orthotopic intracranial model we used cells expressing humanized firefly luciferase. Male 7–8 weeks old nude mice (CD1 nu/nu), 8 mice per group, were anesthetized by intra peritoneal administration of 10%Ketamine (90 µg/gr; Pfizer, Puurs, Belgium) and 2% Xylazine (6.7 µg/gr; Bayer, Brussels, Belgium). Mice were anesthetized with vaporized Isoflurane (1%). Mice were fixed in a stereotactic frame (Kopf Instruments, CA, USA) and a 1.5 cm (longitudinal) incision was made and a burr hole was drilled through the skull at 1.0 mm lateral and 2.0 mm posterior from the Bregma. Tumor cells were injected slowly into the brain cortex to a depth of 2.5 mm with a 22 gauge syringe (10 µl; Hamilton, Bonaduz, Switzerland). After injection, the syringe was left in place for additional two minutes and then slowly retracted. The incision was closed with stitches. Stereotactic challenge was performed under sterile conditions. The mice were weighted, and their health and behavior was evaluated, every two days following the procedure. Greyscale images were acquired at low light with an exposure time of 30 seconds. Bioluminescence images were acquired in the dark using an exposure time of 10 minutes. For analysis of luminescence intensity we used the Signal Meta Vue 6.1 software package (Universal Imaging Corporation). Pseudo-colored overlay images of bioluminescence signal intensity and the mice were generated using the VCTEO2DImageAnalyze software. At the end of the experiment mice were euthanized, the brain was extracted, fixed in formalin, and embedded in paraffin. Prior to fixation brains were cut perpendicularly at the injection site and photographed. All animal experiments performed in Germany complied with the German animal welfare act and were approved by the responsible authorities. Similarly, all the animal experiments performed in Israel were approved by the institutional animal welfare committee and complied with NIH guidelines.

### Immunohistochemistry

Tumors were embedded in OCT and frozen in 2-methylbutane cooled by liquid nitrogen. They were then sectioned into 20 µm thick sections using a cryostat. Sections were fixed with cold acetone, blocked with 10% BSA in PBS containing 0.2% triton, reacted with an antibody directed against CD-31 followed by Cy3 tagged secondary antibody, counterstained with DAPI and photographed using a fluorescent microscope. Eight different microscopic fields derived from two different tumors were photographed. These photographs were taken from areas in which the density of blood vessels was highest (hot spot method) [29], [30]. The area of the blood vessels in fields of equal area was quantified using the NIS Elements BR imaging software (Nikon).

Paraffin sections from brain tumors (4 µm) were antigen retrieved by boiling in citrate buffer (pH-6), treated with 3% H_2_O_2_, blocked with 2% BSA (in PBS) and incubated with anti von-Willebrand-Factor antibody overnight at 4°C. Following washings, slides were incubated with an Alexa Fluor 594 labeled goat anti-rabbit antibody. Nuclei were staining using DAPI staining (Sigma-Aldrich). The samples were mounted with Mowiol 4–88 (Calbiochem) and photographed using a confocal microscope. The image generation and analysis were performed using the Leica Confocal Sofware 2.6 1 Build 1537.

### Chorionallantoic membrane assay (CAM)

Fertilized chicken eggs (spf eggs, Charles River Laboratories, Avian Products and Services, Sulzfeld, Germany) of the domestic fowl (*Gallus domesticus*) were incubated at 37°C and 75% relative humidity. Eggs 10 days after fertilization were kept as recipients of tumor cells. Each egg was candled and the blood vessels in the CAM were marked with a pencil. The egg shell was cleaned with 70% ethanol and a window of about 1 cm^2^ was cut into the egg. The window was sealed with cellotape and the egg returned in the incubator for another week. 7 days later Thermanox tissue culture coverslips (Miles Scientific, Naperville) were placed on the top of the chorionallantoic membrane. Either 20 µl with 1×10^7^ U373MG-NSPI cells in medium, U373MG-sema3D cells in medium or PBS were placed on the discs. 7 days later the CAM was fixed in 6% glutaraldehyde for 10 min. The CAM containing the tumors were excised carefully and pictures from tumors and blood vessels were taken.

### Soft-agar colony formation assay

A layer of agar containing 2 ml of 0.5% low melting agar (Bio-Rad) dissolved in growth media was poured into wells of a 6 well cell culture dish and allowed to set at 4°C for 20 minutes. A second layer (1 ml) containing 0.3% of low melting agar dissolved in growth media containing cells (2×10^3^ cells/ml) was placed on top of the first layer and allowed to set at 4°C for 20 minutes. Each cell type was seeded in triplicates. Growth medium (2 ml) was added on top of the second layer and the cells were incubated in a humidified incubator at 37°C for 21 days. Medium was changed twice a week. Two independent experiments were performed. At the end of the experiment, colonies were stained for 1 hr with 0.005% crystal violet, and incubated with PBS overnight to remove excess crystal violet. The colonies were photographed and colonies with a diameter of at least 100 µm were counted. The average number of colonies per well was calculated and statistics were performed using the GraphPad Prism software.

### Statistical analysis

All the in-vitro experiments were repeated at least three times. In-vivo tumor development experiments were repeated at least twice using at least 7 animals per group. Statistical analysis was performed using the unpaired data with unequal variance student's T-test. Error bars represent the standard error of the mean. Statistical significance is presented in the following manner: *p<0.05, **p<0.01 and ***p<0.001.
